# Understanding gambling in the United Kingdom: A qualitative study on the experiences of gamblers’ families

**DOI:** 10.3389/fpsyg.2023.1009923

**Published:** 2023-03-06

**Authors:** Ferid Azemi, Merita Avdyli, Vilard Bytyqi

**Affiliations:** ^1^Kosovo Academy for Public Safety, Vushtrri, Kosovo, Albania; ^2^Successful Mothers, London, United Kingdom

**Keywords:** addiction, abuse, depression, criminal activity, family relations

## Abstract

This study focuses on understanding the experiences of family members of problem gamblers in the United Kingdom and the gambling consequences in their lives. Family members of problem gamblers, even though they suffer from gambling consequences, the impacts of gambling are under-researched. Qualitative research through in-depth interviews was employed. Thematic analysis was used to gain a deeper insight into gambling. Nine female participants were interviewed based on a semi-structured questionnaire. The results of this study indicated that close family members of problem gamblers had suffered not only financial devastation but also harsh psychological and mental health damages. Furthermore, family members suffer from gamblers’ abusive and violent behaviors. Addiction was a key factor of gamblers’ habit; fear, anxiety, and depression were some of the health issues derived from gambling. Overall, gambling addiction leads to further addiction, crime, and severe, family-relation sufferings. The study results suggest that the London Gambling Commission and regulating authority should change gambling policy, reduce gambling points, and limit gambling slot machines in food markets and community areas. More support is needed for family members affected by gamblers’ addictive gambling behaviors.

## Introduction

Gambling is a widespread social and economic phenomenon that has created as much profit for gambling businesses as it has great personal and financial losses to individual levels around the world. Many people have lost their financial savings and their current financial income due to gambling addiction. Gambling addiction should be treated as a social and health issue today. According to Petry et al. (2017), gambling problems have in the last decade affected 0.2% to 4.0% of the population. The gambling disorder has grown in recent years (Ledgerwood, 2020). Gambling can cause health issues like drug and alcohol abuse (Parhami and Fong, 2015; Matheson et al., 2019; Obedzinski et al., 2019; Reid et al., 2020), and gamblers can be very abusive (violent behaviors) toward their family members (Lane et al., 2016; Palmer du Preez et al., 2018; Hellberg et al., 2019; Suomi et al., 2019). Family members of problem gamblers are more likely to report mental health problems, emotional disturbances, poorer health, and impaired social life (Dowling et al., 2016; Sanchez et al., 2019). The United Kingdom has the tenth highest gambling expenditure *per capita* in the world, equating to about £200 per adult per year (Atherton and Beynon, 2019).

This study is focused in the United Kingdom, where gambling and its social impacts are increasing. Gambling has caused many severe financial issues for many families and, other side-effects. This study explores the extent of suffering and the consequences on family members of the addicted gamblers. Gambling is examined through the life experiences of immediate family members, not the gambler. Neither does the study look at gambling as an industry nor does it examine the statistics related to gambling and its impact on family members. Current research shows that more than half of gamblers face some mental health risk with their gambling habits (Hing et al., 2014). In the U.K., 45.8% of problem gamblers reported being involved in some type of physical fight in the past 5 years compared to 28.0% of non-problem gamblers and 19.1% of non-gamblers (Roberts et al., 2016).

Through in-depth interviews of gamblers, Miller et al. (2018) found that gamblers felt that gambling was a safe environment and entertaining, although they also thought that personal responsibility was important. However, they thought a stigma existed for those gamblers asking for help. According to Quinn et al. (2019), the global gambling market is expanding and expected to reach $635 billion by 2022, largely because of the proliferation of smart devices delivering gambling products. According to statistical data taken from Gross Gambling Yield (Lange, 2020), in Great Britain, the gambling industry increased from roughly 8.4 billion British pounds in 2011 to approximately 14.4 billion in 2018. Furthermore, the gambling industry in 2018 also provided jobs for some 100 thousand employees in the U.K. (Lange, 2020). Statistical data indicated that gambling remains a serious problem and is at once both a healthcare issue and public health concern that affects between 0.12 and 5.8% of the general population worldwide and up to 7% in certain studies (Matheson et al., 2019). According to Parhami and Fong (2015), nearly 4% of the population maintains a gambling-related problem, and 6% will experience harm from gambling during their lifetimes, including financial, legal, relational, and health problems. From this perspective, if the gamblers have families, the scope of harm is wider than the statistical data show.

Gambling not only has caused psychological effects on addiction but also harms society financially. How does gambling impact society financially? Are these the social costs of things like addiction counseling and family welfare? How much profit does the government get from gambling? Is this amount offset by the social costs? Based on the National Gambling Impact Study Commission: Final Report (1999), the amount that customers owed when they walked out of the casinos exceeded $108 million or 20% of the debt. Thus, people getting themselves in debt and not being able to pay their loans is another issue related to gambling. While certain harms caused by gamblers are financial (Parhami and Fong, 2015; Quinn et al., 2019), at the same time, harms extend to personal hurt and damage to interpersonal and family relations (Hing et al., 2014). Family problems and violence are further consequences of gambling-related problems that directly affect relationships (Dowling et al., 2016; Landon et al., 2018; Rodda et al., 2019). Gambling has caused family issues, reflecting gamblers neglecting their children, focusing instead only on their gambling behaviors. Gambling addiction has led to both family breakdowns and financial bankruptcy.

### Research questions

Research questions were created to instigate a deeper understanding of the phenomenology of gambling and its consequences on families in the U.K.

RQ1: What experiences have immediate family members had with gamblers in the U.K.?
RQ2: What impact had gambling problems had on close family members of gamblers?

#### Assumptions

Previous research has determined that gambling is causing family problems. However, gambling as a social and economic phenomenon has thus far not been explored sufficiently in the U.K. The following assumptions will be evaluated:

- Certain researchers indicate that gambling had an impact on family relationships because the impact seems to be negative.
- It is assumed that the planned behaviors of addicted gamblers had an impact on further addiction.
- Immediate family members also feel emotions, including suffering, because of gambling, and this study will bring light to understanding what family members feel and experience.

### Review of literature

To understand the gaps in the literature, we used EBSCO, ProQuest, Google Scholar, Sage, and Scopus to find various credible journals relevant to the topic.

#### Key concepts

Gambling is not only a habit that can lead to addiction but also a healthcare issue (Lane et al., 2016; Palmer du Preez et al., 2018; Hellberg et al., 2019). In this section, gambling is examined for social, psychological, and healthcare issues. According to Matheson et al. (2019), the problem of gambling is a serious public health concern that disproportionately affects people experiencing poverty, homelessness, and multimorbidity, including mental health and substance use. Problem gambling is also a hidden health concern, especially among people who experience poverty (Woodhall-Melnik et al., 2019). This study will shed light on a few principal concepts, such as healthcare and the social harms caused by gambling.

##### Problem gambling family impacts

In a study about the impact of gambling using Problem Gambling Family Impact Measure, using a sample of 212 treatment-seeking problem gamblers in Australia, Dowling et al. (2016) found that 54.7% of the sample indicated substantial unpaid debt and financial deprivation, and nearly half of them or 45.3% of participants reported issues of depression, sadness, distress, anxiety, and guilt. From this study, the loss or damage relationships were part of issues family members suffered. Based on study results, 18.4% of participants suffered damage to relationships with a range of family members, including intimate partners, children, and parents, due to gambling (Dowling et al., 2016). Examining barriers to treatment, using qualitative methods with sample of eight female problem gamblers, Kaufman et al. (2017) found that external barriers such as waiting time for help, waiting for treatment, sense of losing time, and external factors such as denial of the problem, fear, stigma, and ambivalence impacted the gamblers.

Using qualitative methods to investigate the role of family and social situation of 25 female frequent internet gamblers, Corney and Davis (2010), found that most of participants gambled for fun. However, results of this study indicated that long hours spent on gambling impacted the quality of work and damaging impact on the lives including family relationships and finances (Corney and Davis, 2010). In another study about the treatment approaches of family members using mix-method design with 62 family members, Rodda et al. (2019) found that 50% of participants requested gambler-focused options such as advice and support on getting the gambler to change and supporting behavior change, and 28% requested improving the quality of the relationship and managing the impact of gambling. Furthermore, overall, all the results of the study suggested the intervention needed to accommodate both gamblers and their family members (Rodda et al., 2019). A similar study was conducted in Finland using qualitative content analysis of 40 partners of gamblers by using an online discussion forum, Järvinen-Tassopoulos (2020), found that partners of gamblers had to cope with mixed feelings toward the gamblers, some wanted to give their families a chance, whereas others were concerned about their children’s well-being and decided to divorce the gambler. The result of this study indicated that gambling impacted the partners in a variety of forms, such as debt, fear and stigmatization, and trust issues among partners (Järvinen-Tassopoulos, 2020).

In evaluating the harm associated with problem gamblers toward family members, using quantitative methods by using an online panel of gambling problems (*N* = 3,076) and panel members who indicated that they had been affected by someone else’s gambling (*N* = 2,129), Goodwin et al. (2017) found that both gamblers and affected others most often identified close family members, including spouses and children, as the people impacted by others’ gambling problem. The study suggested that there was an estimation of six people (family members) affected by a gambler (Goodwin et al., 2017). Similarly, Dowling et al. (2009), analyzing male pathological gamblers and their female spouses or partners using survey data, found that both the female pathological sample and their partners reported poor adjustment in their dyadic relationship. However, the results revealed substantial dysfunction in the intimate and family relationships of female pathological gamblers, partners, and children of female pathological gamblers (Dowling et al., 2009).

A study conducted by Landon et al. (2018) through a qualitative method, using interview design through semi-structured interviews with a sample of 10 people seeking support through a social agency that reported being affected by someone else’s gambling, found that harmful gambling across generations and the lack of any positive aspects of gambling affected others such as family members and children. The results of the study indicated that gambling had a negative impact on family members, such impacts include family relationships where all participants expressed concern about the welfare of their children (Landon et al., 2018).

In a similar study about the impact of problem gambling on female spouses in Hong Kong, using a qualitative method of 23 semi-structured interviews (all-female participants), Kwan et al. (2020), identified nine domains of impact on participants, (1) spousal relationship, (2) mother–child relationship, (3) relationship with parents-in-law, (4) relationship with parents, (5) financial resources, (6) cognitive-affective functioning, (7) social status, (8) personal assumptions, and (9) sense of making of life. Furthermore, the results of this study indicated that female partners or ex-partners of problem gamblers identified psychological distress, fair or poor health, and fair or poor quality of life (Kwan et al., 2020).

##### Abusive behaviors

In the U.K., 45.8% of problem gamblers reported being involved in some type of physical fight in the past 5 years compared to 28.0% of non-problem gamblers and 19.1% of non-gamblers (Roberts et al., 2016). Many problem gamblers abuse their family members because upon getting addicted to gambling, they begin substance abuse, leading to further abuse toward family members.

In a recent study using a quantitative method, using 212 treatment-seeking problem gamblers, about the violent behaviors in relation to gender differences, Suomi et al. (2019) found that over half (60.8%) of participants reported some form of violence in the past 12 months with no gender differences. Furthermore, the results of this study indicated that the violence was mostly verbal; however, considerable rates of physical violence also were featured in the responses. Since the study was quantitative, there is a gap in knowledge about the perspective of family members suffering from problem gamblers. In a similar study using a sample of 167 male pathological gamblers with a mean age of 39.29 years and 107 non-gamblers with a mean age of 33.43 years, Jauregui et al. (2016), found that pathological gamblers had difficulties of emotion regulation, drug and alcohol abuse, and anxious and depressive symptomatology. Thus, the pathological gamblers posed abusive risks to their family members due to their lack of emotional control and extensive substance abuse (Jauregui et al., 2016). The same results derived from the study conducted by Roberts et al. (2019), using a sample of 1,226 treatment-seeking gamblers, using a survey index, where gambling and criminality are related.

In a study about the evaluation of family violence rates among treatment-seeking problem gamblers compared to general population estimates in Spain, Canada, and Australia, using a sample of 454 problem gambling help-seeking, Palmer du Preez et al. (2018) found that family violence victimization among a sample of New Zealand help-seeking gamblers was estimated at 46.8%, and there is an association between problem gamblers and the violence they use toward their family members. Furthermore, the results of the study indicated that the most common type of violence reported by gamblers and affected others was verbal violence-insulting and screaming at-talking place between current or ex-partners (Palmer du Preez et al., 2018). Most of the studies are focused on quantitative designs that lack a deeper understanding of lived experiences of close family members in the U.K. Similarly, a study investigating characteristics of treatment-seeking problem gamblers, using a sample of 126 participants, to answer self-report questionnaires about gambling behaviors, impulsivity, substance abuse, emotional dysregulation, illegal activities, and gambling consequences, Reid et al. (2020), found that substance and alcohol abuse gamblers had higher levels of impulsivity. However, gamblers of both groups of participants, help-seeking gamblers with adults and non-adults, responded similarly to items on gambling consequences that gambling-related consequences such as struggling to meet the financial obligation and unwanted financial or emotionally hurting a loved one was very high. Overall, the study yelled some interesting findings of impulsivity, and it was found common to both groups of populations (Reid et al., 2020). Since the study was conducted in the U.S., and the methodology was quantitative through self-report questionnaires of those that sought help at clinical centers, there is limited understanding of abusive behaviors of problem gamblers toward their family members in the U.K. In a similar study using a sample of 81 treatment-seeking problem gamblers to explore how a history of victimization, history of family violence impacted gambling behaviors in Australia, Lavis et al. (2015) found that gamblers interviewed indicated that there was no fundamental, statistically significant difference between those problem gamblers with history of victimization only, perpetration only, or no history of family violence. The results of this study indicated that there was a statistically significant finding related to hazardous drinking; male problem gamblers were significantly more likely to display higher levels of alcohol misuse compared with female problem gamblers (Lavis et al., 2015).

##### Genetic and environmental factors

Gambling environments may contribute to planned behaviors of the individuals, which may be predisposed to gambling. Vitaro et al. (2019), using a sample of 373 pairs of monozygotic and dizygotic twins, found that gambling and substance use share a common genetic basis in late adolescence. In a quantitative study using a bivariate model to investigate the magnitude and correlation of genetic and environmental contributions to a gambling problem using a sample of 7,869 male twins in the U.S., Xian et al. (2014) argued that the greater contribution of environmental factors to the co-occurrence between problem and pathological gambling, and milder forms of drug use, suggest that environmental interventions may help to diminish the relationship between problem gambling, and tobacco and cannabis use disorders.

In a qualitative study of gambling harms by Baxter et al. (2019), using secondary data from 1,424 articles published from 2008 to 2017 and relating to regulatory environments in Canada, Australia, and New Zealand, it was found that countries with privately-operated gambling focused on harm factors that fell under the responsibility of the operators. At the same time, jurisdictions with a public health model focused on treatment and harm resources. Adams and Wiles (2017) conducted a study about gambling in New Zealand argued that the proliferation of commercial gambling machines had contributed to a global increased in the rates of addictive behavior associated with gambling. Adams and Wiles (2017) concluded that the gambling machine annex is the main enabling space for generating harms, and yet most persons in fact, understand only little about how they function.

A similar study was conducted by Quinn et al. (2019) about self-efficacy to control gambling, and the relationship between the perceived impact of gambling advertising found that significant interactions between the subscales of gambling involvement and awareness and self-efficacy to control gambling, on problem gambling severity. Thus, the results suggest that the high perceived impact of gambling advertisements on involvement and awareness, but not knowledge, relates only weakly to high levels of problem gambling.

Wu and Chen (2014) conducted a study comprising a sample of 416 residents of Macau and 409 from Singapore, found that people accept every benefit from casino gambling as an outstanding choice but unavoidably encounter its negative impacts. Wu and Chen (2014) concluded that community perception varies in relation to casino development, and positive perceptions are related to tourism attraction.

In a longitudinal interview study using a sample of 596 young U.S. adults, Martins et al. (2013) found that one-third of the sample of 187 participants were past-year gamblers, 42% of them gambled more than once a week, and 31% had gambling-related problems. Furthermore, Martins et al. (2013) argued, social and environmental influences on gambling behavior are important to understand because localities can control the sanction and location of gambling opportunities. In this context, the authors argued that higher neighborhood disadvantage was associated with gambling frequency and problems among young adult gamblers from an urban, low-income setting (Martins et al., 2013).

Casino availability and the impact of gambling, using data from a representative sample of 50,048 Canadians across four provinces, Philander (2019), found that increased exposure to casinos is correspondingly related to increases in both gambler participation and problem gambling risks, despite the observation that all four provinces recently experienced casino expansion and population-wide declines in problem gambling prevalence rates. Salonen et al. (2018), conducted a study using a sample of 7,186 persons, including a clinical sample of 119 in Finland through a survey design, found that that man gambled more than women, and the most common gambling environments were kiosks, grocery stores, and supermarkets. Another issue with gambling is the online system of casinos, where about 20% of gamblers had played videogames and betting for money (Stark et al., 2020).

Harris et al. (2018) conducted research using 70 regular (non-problem) gamblers, by investigating the potential to induce impulsivity transfer effects within an electronic gambling context found those non-problem gamblers demonstrated that structural modifications to slot machine gambling could affect executive control domains. These domains included motor response inhibition and delay discounting, as well as information sampling.

Using a sample of 1,978 gambling venue patrons over the age of 55 years in Canada, using U-test bivariate analyses, van der Maas et al. (2017) found that past-year bus-tour patronage was associated with more frequent slot machine play. Furthermore, van der Maas et al. (2017) also concluded that adjusting for potential confounders among older adults using bus tours access Canadian gambling venues is associated with an increased risk of problem gambling. Availability and tours around gambling venues, the authors concluded, were associated with gambling.

##### Health and social harms

In survey research using quantitative design, Salonen et al. (2018) argued that the most common gambling-related harms were financial or emotional/psychological ones. Using a clinical sample of 119 participants, Salonen et al. (2018) found that the most common reason for gambling was the chance of winning money. Another argument determined by Salonen et al. (2018) from the study in Finland was that men gambled more often than women for excitement, entertainment, and fun, while women gambled more often than men to win money.

In the study about gambling disorder, using a meta-analysis of 11 population surveys, Parhami and Fong (2015) found that higher mean prevalence for nicotine dependence (60.1%), a substance use disorder (57.5%), mood disorder (37.9%), and anxiety disorder (37.4%). Furthermore, based on their longitudinal three-year study, Parhami and Fong (2015) concluded that any mood, anxiety, or substance use-related disorder was more likely to develop in individuals with either subthreshold gambling disorder or actual gambling disorder than it did in those persons who did not gamble.

In a similar study using a sample of 1,259 Indigenous Australians, using the Problem Gambling Severity Index, Hing et al. (2014) argued that harms include financial difficulties and feelings of guilt and regret about gambling. Hing et al. (2014) found that most problem gamblers relied on family, extended family, and friends for financial help or went without them as a direct result of gambling losses. In this study, Hing et al. (2014) concluded that nearly half of the sample did not think they did, in fact, maintain a problem with gambling, but the results showed that the majority or 57.7%, did fact experience at least some risk because of their gambling habits.

Jonsson et al. (2019), trying to answer the questions about the relationship between gambling disorder and public health issues, using a randomized controlled trial design to evaluate behavioral feedback, argued that participants assigned to the phone calls or letters over 12 weeks, contacting high consumers about their gambling expenditure appeared to be an effective method for gambling companies to meet their duty of care to customers.

Using a survey design with 283 participants (students) in Canada, Obedzinski et al. (2019) argued that increased problem gambling risk was associated with higher levels of alcohol-related problems, such as binge drinking, cigarette smoking, use of illegal drugs, and unplanned and unprotected sex. Similarly, Columb et al. (2018) conducted a research survey of gambling disorder processing from health centers in Ireland, and these surveys were sent to medical centers across Ireland to see if these centers offer gambling disorder assistance. The findings from this study indicated that some community health centers offered services to gambling disorders; however, some centers did not offer any kind of gambling disorder help (Columb et al., 2018).

##### Summary of literature review

Most of the studies are quantitative, and not many studies are conducted about family members. There is a gap in literature because most of the previous studies focused on clinical groups such as treatment-seeking gamblers or survey data delivered to health centers that may treat or deal with addictive gamblers.

## Method

Qualitative methodology through interview protocol was employed for this study. The main reason why we used the qualitative method through interview design was to gain focused insight of lived experiences of participants. Also, the interview design is used to understand participants making sense of and construct reality in relation to the phenomenon (Ravitch and Carl, 2016), engagement, and explore gambling issues. There was a challenge in finding interviewees due to their time and not being interested in a study. Initially, 12 participants volunteered (nine females and two males); however, two participants withdrew from the study without any reason; therefore, all participants for the interviews were females.

### Participants and procedure

For this study, a qualitative method through interview design was used. In-depth interviews using semi-structured questionnaire (see Appendix A) were conducted with gamblers’ family members with the purpose of understanding the effects and consequences of gambling. This study was initiated in the London area in October of 2018 and throughout 2019. Before conducting the study, we contacted the U.K. Research Integrity Office for ethical approval (UKRIO 2020–95) and were given the green light to conduct research as long as we follow the ethical guidelines. Initially we posted a flyer online for interested participants in this study. The sampling technique selected consisted of snowball sampling, in which participants declared reference to other persons with similar issues related to gambling. Nine participants, all women, were interviewed. Sample selection criteria included any person who is an immediate family member of a gambler, who was an adult, above 18 years of age, willing to be interviewed voluntarily, and who was willing to talk about gambling. Participation was voluntary, and during the study, any participant could discontinue an interview at any time and for any reason. Before interviewing subjects, researchers explained the purpose of the study to each participant separately. All interviews took place separately in a series of quiet restaurants in London. The reason why the interviews were conducted in a quiet place in a restaurant is due to the request made by participants. Each participant was provided with an informed consent form emphasizing that the interview was voluntary and that subject withdrawal from the project was possible at any point. Each subject, before the interview, was also required to sign a consent form where she would share their experiences, but her name would not be disclosed to the public. Participants were given permission to leave at any time during the interview and the right not to answer any question.

Each interview was voice-recorded and was transcribed, and from these transcripts, there were generated 80 codes. From codes, four main themes were generated: (1) self-blame, (2) denial of the truth, (3) mental problem, and (4) financial bankruptcy. To ensure that participants understood all questions, we explained each query in a simple way and conducted follow-up questions. After themes were generated from thematic coding—itself a product of careful analysis—the consequent results shed meaningful light on this exceptionally complex phenomenon.

### Coding

Since the study consisted of interview design, all the interviews were voice-recorded, and on average 40 min, each interview lasted. The interviews were returned to transcripts, and then the coding process began. About 80 codes were generated from all interviews. It is apparent that coding is a shortcut for a researcher to get to the point without overloaded words and statements. According to Lapan et al. (2011), labeling codes with gerunds such as controlling, and coping, helps researchers as grounded theorists to remain focused on action and processes as well as to make connections between codes. By coding, the researcher records the essence of the data collected. According to Ravitch and Carl (2016), coding data involves reading for regularly occurring phrases, terms, interactions among actors, strategies and tactics, consequences, and patterns of participation. There are many reasons and justifications why coding is important in qualitative research. Researchers also use coding in order to reduce repeated essence-capturing words or statements. Ravitch and Carl (2016) explained that “the important thing in coding is to engage in ways that resist these troublesome tendencies and engage a systematic and consistent approach to the data analysis” (p. 251). Coding has multiple functions; therefore, applying this process during research is important because it will keep data organized and reduce overloaded data recorded and analyzing. Coding is a process to keep data assessable and also available easier. In this study, codes were manually processed, and NVivo was used mainly for processing the transcripts. For instance, the codes were generated from carefully analyzing the interviews, in an answer by a participant: “I should have prevented my husband from gambling at the beginning, and I am the one to be blamed for this and other gambling habits and problems.” In this case, preliminary codes are: *Preventing gambling* and *blame.*

### Demographics

All participants of this study were closely related to gamblers. They were ex-wives, daughters, and one sister of a gambler (Table 1).

**Table 1:** Participant demographic data.

Code	Relationship to gambler	Age	Gender	Years lived with gambler
LP1	Ex-wife	52	F	19
LP2	Ex-wife	41	F	20
LP3	Ex-wife	45	F	16
LP4	Ex-wife	34	F	9
LP5	Sister	20	F	10
LP6	Daughter	25	F	9
LP7	Daughter	20	F	10
LP8	Daughter	21	F	10
LP9	Daughter	21	F	5

## Results

The qualitative method through in-depth interview design was applied in this study. Each interview was codified, and through thematic analysis, results reflect the emotional and financial issues each participant had endured due to the gambling habits of gamblers. All interviews were subsequently turned into a transcript and codified. Four main themes were generated from codes. Each participant indicated that the gambling of their respective immediate family members had led to enormous problems in their life. Based on participants’ experiences, gamblers began gambling a few years earlier, where none of the immediate family members knew about this. Participants in this study also shared their lived experiences about the gambling consequences, the impact that left them in deep debt and suffering emotionally.

## Discussion

The results of this study are compelling because they brought into light not only family issues deriving from the gambling phenomenon but also the mental health harm and emotional devastation. The following four main themes came out of this study: (1) self-blame, (2) truth-denial, (3) mental health issues, and (4) financial bankruptcy.

### Self-blame

Most of the participants, to a certain degree, blamed themselves for not being able to prevent the gambler from their gambling addiction. Participants LP1, LP2, LP3, LP8, and LP9 all indicated that they blamed themselves for letting their family members gamble, and they felt that they share responsibility for not acting in preventing and stopping gambling of their family members. Participant LP1 stated: “*I blame myself for him (now ex-husband) gambling, I have taken all responsibility in my home, I should have been adamant at the beginning, and I have given him all the freedoms*.” Similarly, Kwan et al. (2020) found that most of the female partners of problem gamblers felt guilt-related gambling issues. Similar findings are confirmed in Hing et al. (2014) study, where most problem gamblers relied on family, extended family, and friends for financial help. Thus, in this case, the participants do self-blame for not noticing this issue earlier. Participant LP2 indicated: *“I blame myself; I did not prevent…. I have closed my eyes; now I only live for my kids, that is all, I have no life.”* Similar findings were brought by Palmer du Preez et al. (2018), with the results indicating that family violence victimization among gamblers and family members and was found an association between problem gamblers and the violence they use toward their family members. On the other hand, participant LP3 claimed: *“We lost a person we looked up to and was wondering how to come around the situation, did not know how to help him.”* Similar results were found in research by Navas et al. (2016), in the study with observed participants showed self-blame and catastrophizing emotional regulation. Participant LP9 pointed out*: “I should have detected earlier, but I was very tolerant. I thought gambling would go away, I blame myself for allowing the situation out of hand.”* This confirms the study results by Roberts et al. (2016), indicating that many problem gamblers abuse their families and break the family trust. Overall, self-blame is more of a sign of psychological difficulty in coping with reality. The results of the study also confirm the findings by Kawan et al. (2020), where female family members were impacted on relationships with problem gamblers and the impact on spousal relationships, including trust, communication, and intimacy.

### Truth-denial

The attempt to convince a gambler is another theme that most of the participants mentioned during interviews, which has a meaning that when participants are faced with a gambler, in all cases, the gambler denied gambling, trying to hide from the truth. All participants of the study dealt with acknowledging the gambling problem and the subsequent seeking of the truth. Participant LP3 indicated: *“Initially, he was in a denial of gambling, and he made a promise that he was helping his cousin and other relatives with money.”* Another participant, LP4 stated: *“He was in complete denial of gambling.”* The results of this study indicated that gamblers, when they spoke with their loved ones about the problem, tried to hide the truth, denying any involvement in gambling. The gamblers also declared that they would never do such a thing. Participant LP7 observed: “*Violence and arguments became very common…. It made hard to trust him; before gambling, I thought he was the best daddy in the world, now I do not feel close to him.”* Similar results were derived in the study by Järvinen-Tassopoulos (2020), where fear, stigmatization, and trust issues were found among partners. Hing et al. (2014) found similar results, indicating such harms as financial difficulties and feelings of guilt and regret and denial about gambling. This also confirms the study results by Jauregui et al. (2016), indicating that pathological gamblers posed abusive risks to their family members due to their lack of emotional control and extensive substance abuse. Participant LP6, LP7, and LP9 stated: *“when faced with the facts of gambling evidence, he became verbally abusive toward me, and threatened me.”* Similar results are found in the studies by Roberts et al. (2019), indicating that problem gambling and family violence were common. What participants stated also is confirmed in a study by Palmer du Preez et al. (2018) that family violence victimization was at a higher rate and very common. All participants tried to help their family members who were addicted gamblers. However, there was a great resistance to admit gambling habits by gamblers. All gamblers tried to lie when their loved ones confronted them; however, they could not leave gambling and return to normal life. Similar results are found in a study by Adams and Wiles (2017), indicating that increased addiction was related to gambling. Each attempt was unsuccessful. This led to divorce and the separation of gamblers from their family members. This is confirmed by study results conducted by Obedzinski et al. (2019), indicating that increased problem gambling risk was associated with higher levels of alcohol-related problems, such as binge drinking, cigarette smoking, use of illegal drugs.

### Mental health issues

One of the main issues that family members faced about gambling is the mental health problems and paranoia that followed them even after they distanced themselves from the gambler. All participants interviewed, to a certain extent, suffered mental health breakdown and emotional devastation. Each interviewee indicated that she suffered from anxiety and fear. Participant LP1 stated: “*It’s affecting my health, I am getting weaker and weaker, it’s like a war zone.”* This is confirmed by study results conducted by Matheson et al. (2019), in which gambling had led to mental health issues, ones that affected both gamblers and respective family members. Furthermore, the same participant described that she felt paranoia, and fear, specifically a constant fear. The emotions of fear, anxiety, and mood disorders were found among gamblers in a study by Parhami and Fong (2015). Another participant, LP2, described the issue of stress and depression from gambling as follows: *“I do not sleep, I cry, live in fear, and in one month I lost five kilos, and now I smoke also. It’s mental health, too much stress; it is always affecting my health.”* Similar results about psychological harm from gambling were found in studies by Salonen et al. (2018) and Parhami and Fong (2015), where, in both cases, anxiety and mood disorders were developed as a result of the gambling phenomenon.

### Financial bankruptcy

In general, the main purpose of gambling is financial gain; however, most of the time, financial bankruptcy is inevitable. All participants, in one or the other way, have suffered bankruptcy and huge financial losses from a family member who was an addicted gambler. Participant LP1 described the gambling effects in a financial context as follows: *“We planned to buy a house, but $65,000 are gone, on gambling. He is $120,000 in debt and never paid rent for three months; he has people he owns money.”* Other participants also related cases to financial issues and bankruptcy. Similar results derived from a study conducted by Reid et al. (2020), indicating that unwanted financial loss or emotionally hurting a loved one was very high. Participant LP2 stated: *“All 15 years, the money he has saved is gone; he did not get any back from gambling.”* Participant LP3 stated*: “I cannot believe how he had done like that because $20,000 are gone on gambling.”* Participant LP4 indicated that her ex-husband sold their house because of gambling debt.

Furthermore, participant LP4 stated: *“He destroyed my life, every morning and every night I live with the fear that someone will knock in the door to ask for money due to gambling.”* This confirms study results conducted by Salonen et al. (2018), arguing that the most common gambling-related harms were financial or emotional/psychological ones. Another participant, LP5, talking about her brother, a gambler, remarked: *“In one night, he lost $12,000.”* Participant LP7, describing her parent, another gambler, claimed: “*He lost his passion for life, lost his job. He started to sell things from the house.”* As earlier researchers have frequently also determined, gambling creates a negative effect on the financial context of gamblers, leaving them in debt (e.g., Petry et al., 2017; Salonen et al., 2018; Philander, 2019).

## Recommendations

Gambling, even though it may bring financial gains to individual businesses, and increase tax revenue at a central level, should still nevertheless be treated as a social issue. Reducing gambling environments such as casinos and slot machines in food markets may decrease the number of gamblers in the larger society. Numerous studies have indicated that gambling is at once social harm and mental health issue (Parhami and Fong, 2015; Woodhall-Melnik et al., 2019). Therefore, local government offices in London and other cities should establish consulting and psychological therapies for gamblers and their family members. Perhaps a campaign about the risk factors of gambling addiction would be a good start. It would be wise to close the gambling points in food markets. Lawmakers must consider changing current gambling policies.

### Implications

Gambling can lead to addiction, and addiction to gambling can, in turn, continue to such other addictive behaviors as drug and alcohol abuse, which later may lead to domestic abuse and violence. Reducing gambling through policy changes would also be considered a significant step toward reducing domestic psychosocial abuses. The community would much rather benefit from keeping its members in a great family relationship rather than divorce and financial bankruptcy because of gambling environments, such as food markets and other easy-access areas. It is recommended that government agencies open professional consultation offices for anyone who endures gambling issues, and thus, families can benefit from the support offered to families with gambling issues. Perhaps establishing a training center to cure gambling addiction through psychotherapeutic programs would reduce this social issue.

### Limitations

Since the study consisted of qualitative design through interviews, the results cannot accordingly be generalized. The results of this study are only for this category of individuals that are affected by gambling consequences. The study is focused mainly on London. Since all participants are females, it is still unknown if gender plays a role in viewing or perceiving gambling in a different dimension. Thus, it is still unknown if other possible participants from other cities or places think or feel similar to the matter. Since all participants were of female gender, it is still unknown if the results would be different with male participants. It is unknown, for gamblers themselves, what kind of experiences they have during their planned behaviors. Further research is needed to answer gambling as a social and psychological issue.

## Conclusion

The results of this study emphasized the bitter experiences of the participants interviewed. This study reflects four main gambling consequences experienced by a family member of gamblers in the U.K.: Self-blame, denial of the truth, mental problem, and financial bankruptcy. Overall, gambling can cause severe financial and psychological damages to a gambler and all family members. All participants have paid a stiff price as a result of gambling addictions by loved ones. Fear, anger, anxiety, and separation from gamblers became part of their everyday lives. Gambling has caused a high degree of stress and devastation and a loss of hope for a better future. Even though participants tried hard to help the gambler, each participant interviewed nevertheless failed to convince the person to leave gambling and return to normal life. Verbal, physical, and psychological abuses became the lifestyle of family members of gamblers. Most of the family members lived in fear that they may be kicked out of home due to the gambling debt in their family. The results indicate that policymakers must develop a strategy to address gambling addiction and increase awareness of the risk factors that can cause gambling addiction. This determination can be considered as a small portion of the requisite understanding of the gambling phenomenon. However, further research is needed to fully understand how many other gambling dimensions, such as crime and violence, are related to gambling.

## Authors’ note

FA is holder of bachelor of Science in Criminal Justice at American Intercontinental University, Master of Science from Boston University, and holds Ph.D from Walden University in Public Policy and Administration with the specialization in Criminal Justice. He is author of many research articles and textbooks.

MA is founder of Successful Mothers platform and community oriented company. MA holds Bachelor of Criminology and Social Sciences degree and she is coauthor of many research articles.

VB is holder of Bachelor degree and Master of Law at University of Pristina. He holds Ph.D in Law at European Southeastern University.

## Data availability statement

The raw data supporting the conclusions of this article will be made available by the authors, without undue reservation.

## Ethics statement

The studies involving human participants were reviewed and approved by UK Research Office. The patients/participants provided their written informed consent to participate in this study.

## Author contributions

FA designed the research proposal and prepared interview protocol. MA carried interviews and set interview appointments. VB contributed in treating legal framework of gambling. All authors contributed to the article and approved the submitted version.

## Conflict of interest

The authors declare that the research was conducted in the absence of any commercial or financial relationships that could be construed as a potential conflict of interest.

## Publisher’s note

All claims expressed in this article are solely those of the authors and do not necessarily represent those of their affiliated organizations, or those of the publisher, the editors and the reviewers. Any product that may be evaluated in this article, or claim that may be made by its manufacturer, is not guaranteed or endorsed by the publisher.

## Appendix A: Semi-structured questionnaire

**Table:**

Semi-Structured Questionnaire—Participant Information Code:
First name and last name of participant: Time and date of interview: Birthdate/ place of birth: Place of interview: Education: Relationship with gambler: Year of knowing your family member is gambling: 1. Describe your current life and your relationship with the person who gambles. 2. When did you find out that your family member gambles? What was your reaction? 3. What was the reaction of the person who gambles when faced with your knowledge of their gambling? 4. In your opinion, what has led the person you know to gamble? 5. What was the financial status pre-gambling of your family member? 6. What is your current financial status since your family member began gambling? 7. What kind of issues and problems did you feel at the beginning stage of the gambling of your family member? 8. To what degree does your family member effects your life? Can you elaborate? 9. What kind of feeling did you acquire when you found out that your family member lost on gambling? 10. How much the gambling situation had an impact on your life and on your future? 11. What were the reactions of your other family members concerning their brother/sister/husband/wife gambler? 12. Did you or other non-gambling family members ever receive threats from gamblers? 13. What was your relationship with the gambler before and after the person began gambling? 14. How do your relatives act when they found out that your family member gambles? 15 What kind of help did you or the family member who gambles receive from the government?
